# Autism and the Menopause Transition: A Mixed-Methods Systematic Review

**DOI:** 10.1177/25739581251369452

**Published:** 2025-09-01

**Authors:** Aimee Grant, Harriet Axbey, Willow Holloway, Selena Caemawr, Monique Craine, Hazel Lim, Sebastian C.K. Shaw, Rebecca Ellis

**Affiliations:** 1 School of Health and Social Care, Swansea University, United Kingdom; 2 Autistic UK CIC, United Kingdom; 3 Brighton and Sussex Medical School, United Kingdom

**Keywords:** Autism, Autistic adults, Autistic women, climacteric, healthcare, menopause, neurodiversity, perimenopause, systematic review, wellbeing, women

## Abstract

**Background:**

The menopause transition commonly occurs between the ages of 45 and 55 years. In a general population, hormonal shifts result in a range of biological, psychological, and social changes. Recently, research has begun to focus on Autistic people’s experiences of the menopause.

**Methods:**

We undertook a prospectively registered (PROSPERO: CRD42023450736) systematic review of research and first-hand accounts from grey literature related to Autism and menopause. We utilised the Joanna Briggs Institute convergent integrated synthesis approach.

**Results:**

Our search identified eight studies and seven pieces of grey literature, primarily comprising Autistic people. No studies evaluated interventions or provided data from those supporting Autistic people. We developed three themes. First, “knowledge of the menopause transition and peer support” focused on Autistic people’s lack of knowledge of menopause symptoms, including differences for Autistic people, and the role of peer support in obtaining knowledge. Second, “Autistic people’s experiences of menopausal symptoms” describes a broad range of negative symptoms which sometimes had significant impacts on mental health and daily activities. Limited quantitative evidence highlighted increased menopause symptom severity for Autistic people compared to non-Autistic comparison groups. Menopause symptoms impacted on work and relationships, and there was an inter-relationship between menopausal symptoms and Autistic identities. Third, “treatment of menopause symptoms” describes non-medical and medical approaches, including Hormone Replacement Therapy, to reduce symptom impacts. Most reports of medical treatment highlighted barriers to access, or negative experiences of appointments.

**Conclusion:**

There is a clear need for better menopause supports for Autistic people. This should include Autism-friendly information to increase knowledge of menopause, and how it may impact Autistic people. Corresponding information should also be available for health professionals, with systemic barriers to healthcare also reduced to allow the best chance for Autistic people to receive menopause support. Autism-specific menopause peer support may be worthy of evaluation.

## Background

The menopause transition (also referred to as ‘perimenopause’ or ‘climacteric’) refers to the body’s transition from reproductive to non-reproductive life in those Assigned Female At Birth (AFAB) and is associated with significant hormonal changes.^1^ During the menopause transition, the length of time between menstrual periods typically increases,^2^ and perimenopause ends in menopause when a person has not had a menstrual period for 12 consecutive months.^3^ Perimenopause most commonly occurs between the ages of 45 and 55, but may also occur earlier.^4^ The length of the menopause transition fluctuates from person to person and typically lasts several years.^3^ Some people may also have their menopause induced earlier due to medical interventions (e.g., ovarian surgery, cancer treatments, gender reassignment), this is known as induced or surgical menopause.

In a general population of AFAB people, the menopause transition is associated with a range of biological, psychological, and social changes.^1^ Symptom severity typically increases in late perimenopause,^5^ and may continue following menopause.^6^ Genitourinary symptoms affect around half of menopausal people; these include physical changes to the genitals (urogenital atrophy), vaginal dryness, vaginal itching (pruitus), incontinence and bladder problems, and pain during sexual intercourse.^7^ Vasomotor symptoms, including hot flushes and night sweats, are also common, affecting over half of those going through the menopause, and may result in sleep disturbances and insomnia.^5^ Other common menopause changes include: psychological symptoms (anxiety, depression, mood fluctuations, loss of libido), and cognitive symptoms (problems with memory and concentration, headaches, fatigue, irritability).^8^ These symptoms are experienced to varying degrees in a general population, with some people experiencing ‘severe and disruptive symptoms’, and others experiencing symptoms considered to be ‘mild’. ^9(pp1-2)^ However, there is variation in experiencing menopause symptoms by ethnicity, with some ethnic groups within Asia reporting fewer symptoms,^10^ and this is at least in part related to the presence of soy – a source of phytoestrogens – in the diet.^11^

The gold standard treatment for menopausal symptoms is Hormone Replacement Therapy (HRT), including oestrogen, progesterone, and testosterone, which is known to reduce genitourinary and vasomotor symptoms.^5^ There are a range of doses and delivery mechanisms available for HRT.^12^ Whilst there is some evidence that health professionals (in both primary and tertiary care) feel confident treating menopausal symptoms^13^ those who have sought medical help for the menopause report a range of issues with care, including being dismissed by health professionals, lack of health professionals’ knowledge, and lack of effective treatment.^14^ Furthermore, those going through the menopause may also use complementary and alternative therapies, which currently lack evidence of effectiveness.^13^ This may be influenced by social media content, which currently is not aligned with the evidence-based menopause care.^15^ Higher levels of family support has also been associated with reduced menopause symptom severity in China.^16^ However, in UK-based research, support from partners was felt to be lacking, with social support provided by others with lived experience of the menopause.^17^ There are also reports that listening to others’ menopause stories can help to increase menopause knowledge and reframe thoughts relating to menopause symptoms.^18^

Autistic^*^ people have worse mental and physical health outcomes than non-Autistic peers^20^ and a lower life expectancy.^21^ They are more likely to experience adverse childhood events^22^ and traumatic experiences.^23^ Moreover, Autistic people can also have greater difficulties dealing with life changes, and experience life events as more stressful than non-Autistic people.^24^ Autistic people have different communication styles compared to non-Autistic people. In the context of a society which expects neurotypical communication, this may reduce the support available from health professionals,^25^ and through social relationships leading to feelings of isolation and loneliness.^26^ In addition, most Autistic people experience difficulties processing the sensory world and difficulties with interoception.^27^ These known differences are likely to affect their experience of the menopause transition and may account for a recent finding that only 4% of Autistic women aged 46-70 years had recorded menopause symptoms in their medical records; half of the rate of a general population.^28^

## Methods

Our systematic review was undertaken, and is reported, following the Preferred Reporting Guidelines for Systematic Reviews and Meta-Analyses (PRISMA).^29^ We also followed the Joanna Briggs Institute (JBI) mixed methods approach.^30^ Our review was prospectively registered with PROSPERO (registration ID: CRD42023450736).

### Aim

To undertake a mixed methods systematic review of the menopause-related experiences and needs of Autistic AFAB people.

### Research team

This systematic review is being undertaken as part of a larger research project which aims to explore the experiences of Autistic AFAB people from menstruation to menopause,^31^ to generate new knowledge, and to drive improvements in reproductive healthcare for Autistic people. This review is Autistic led (by AG) and was undertaken by an entirely Autistic academic research team (RE and HA). Furthermore, four Autistic adults with lived experience of the menopause transition (WH, SC, MC, and HL), and an Autistic primary care doctor (General Practice Registrar, SS) were involved in the analysis and writing up of this review. All authors contributed to developing the discussion.

### Searches

Autism related search terms were generated from reviews of Autistic adults’ experiences of other areas of life.^32,33^ Menopause related terms were generated through discussions with an Autistic primary care doctor (SS) and drawing on the search terms of menopause-related systematic reviews.^34–36^ Our search terms related to (1) Autism and (2) Menopause:

Autis* OR “Autism Spectrum Disorder*” OR ASD OR ASC OR “Autism Spectrum Condition*” OR Neurodiver* OR “Autistic Disorder*” OR “Autism Disorder*” OR “Child Development Disorder*” OR “Child Development Disorder (adj 2) Pervasive” OR Asperge* OR “Asperger Syndrome*” OR Neurodevelopment* OR “Neurodevelopmental Disorder*” OR “Neurodevelopmental Condition*”

AND

Menopaus* OR Premenopaus* OR Pre-menopause* OR “Pre Menopause” OR Perimenopaus* OR Peri-Menopaus* OR “Peri Menopause” OR Postmenopaus* OR Post-Menopaus* OR “Post Menopaus*” OR “Pre-Menopausal period” OR “Pre Menopausal Period” OR Climacteric OR Climacteri* OR “Surgical Menopaus*” OR “Induced Menopaus*” OR “Oopherectomy” OR “Cessation of Menstruation” OR “Vasomotor” OR “Hot flashes” OR “Hot flushes” OR “Primary Ovarian Insufficiency” OR “Ovarian Insufficiency” OR “Ovarian Failure” OR “Hormone Replacement Therapy” OR “Hormone Replacement” OR “HRT” OR “Amenorrhoea” OR “Amenorrhea”

Four databases were searched for relevant literature (Medline via EBSCOhost, APA PsycINFO via EBSCOhost, CINAHL via EBSCOhost, Web of Science via Clarivate). Searches were limited to humans, from 2003 to present and were conducted in August 2023. We included literature spanning a 20-year period, to balance the relative scarcity of literature whilst also ensuring relevance. Citations were saved into separate Endnote libraries for each database, as per PRISMA guidance,^29^ and screened for duplicates. Additional searches included forward and backward chaining of included studies.

We also undertook internet searching for grey literature. This included using the terms related to “Autism” and “menopause” (noted above) within Google in March 2024. Each item identified in the search results was evaluated systematically for their relevance to Autistic experiences of the menopause transition. When two consecutive pages of search results had returned no on topic results, that search was ended.

### Study selection and eligibility criteria

We used the ‘population’, ‘context’, ‘phenomenon’ approach to inclusion and exclusion criteria, which is narrower than is typically used in JBI reviews,^30^ to maximise relevance. Studies were considered against the inclusion and exclusion criteria in Table 1. Grey literature relating to these terms, which did not report on an already included study, was also included.

### Screening

The title and abstract of papers were independently screened by two researchers (RP and HA). The full text of potentially relevant studies were then reviewed independently by two researchers (AG and RE). Any disagreement was resolved through discussion, and it was not necessary to include a third reviewer.

### Quality assessment and data extraction

Data extraction was undertaken by AG, comprising demographic and methodological details (as seen in Tables 1 and 2) as well as all relevant content on the review’s focus, including from the abstract and appendices. The peer reviewed research studies were independently subjected to JBI quality appraisal by AG and RE, using both the cross-sectional and qualitative tools; disagreement was resolved through discussion.

### Synthesis approach

We followed the JBI mixed methods guidance, using a convergent integrative synthesis approach.^30^ This meant that quantitative data were transformed into qualitative data by converting quantitative data into ‘themes, categories, typologies, or narratives’.^37(p2112)^

All data were imported into NVivo (R1) and coded line-by-line by AG. AG prepared a full draft and summary of the initial analysis, as well as preparing an electronic white board (using Miro.com) with moveable “sticky notes” displaying themes, sub-themes and adding discussion points. Two two-hour data analysis meetings were held in the Autumn of 2024 between AG and the lay authors (WH, HL, MC, SC), with a further 2-hour meeting between AG and the academic authors (RE, HA, and SS), to allow the development and refinement of the themes presented. The final themes were discussed and agreed upon by all authors.

## Results

### Studies identified

**Figure 1:**
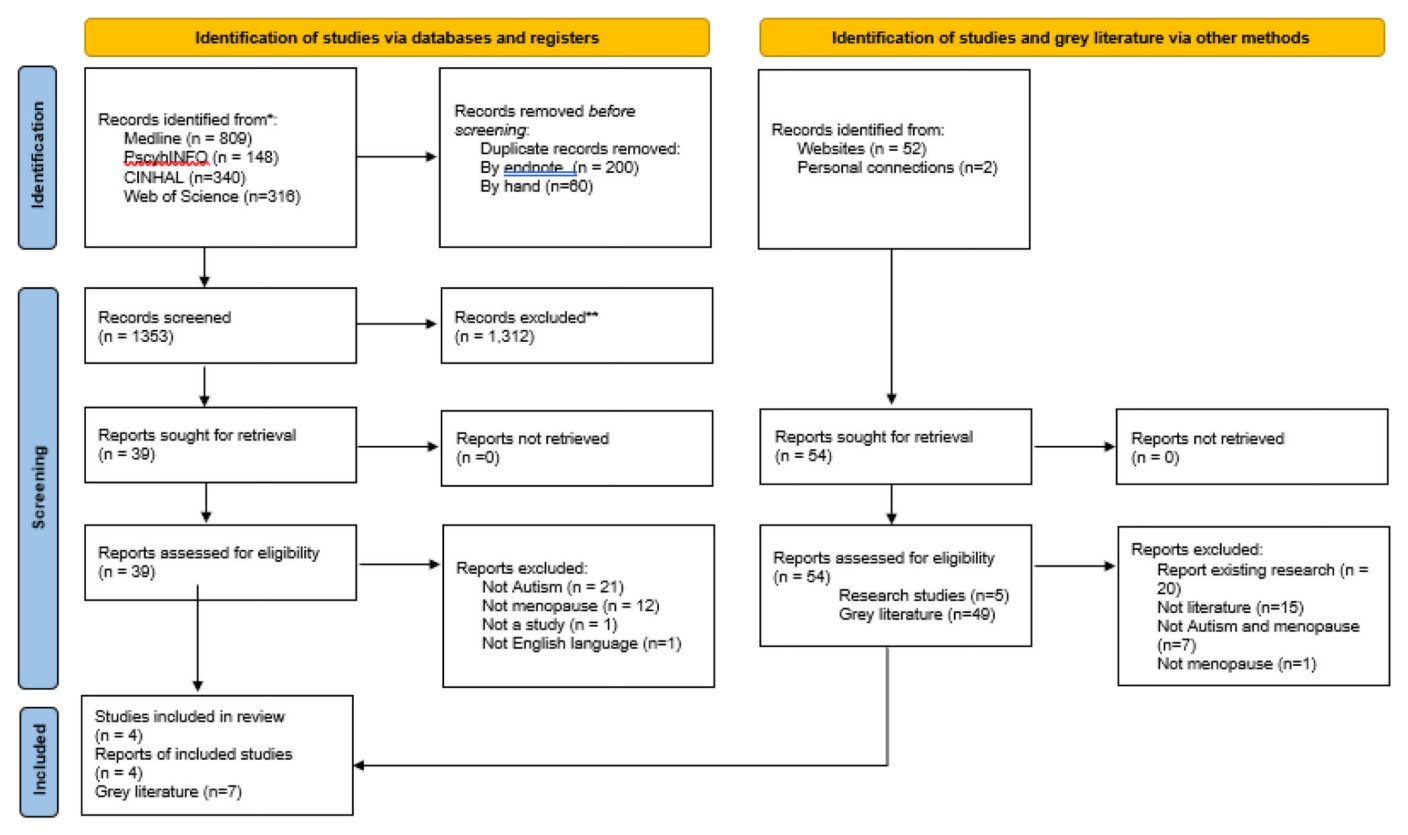
PRISMA flow diagram

Database searches identified 1,353 unique records (see Figure 1), 39 of which were fully screened against the inclusion criteria with four included (see Supplementary information 1 for exclusion decisions). Online searching and personal connections identified 54 further records which were screened. We included seven pieces of grey literature. Furthermore, three additional research studies^38–40^ and one pre-print^41^ were published following our database searches and included. Whilst two were included before the thematic synthesis began,^38,41^ two were added once the thematic synthesis was in progress.^39,40^ One systematic review^42^ was unpicked and included articles were subjected to forward and backward chaining, but no new studies were identified. In total 15 sources, eight studies and seven pieces of grey literature, were included.

Table 2 shows that half of the eight included studies were qualitative in nature, using interviews (n=2), focus groups (n=1), or both (n=1). A further four studies were cross sectional, including assessing menopause symptoms quantitatively (n=2), a mixed methods survey including content related to healthcare consultations for menopause (n=1), or both (n=1). The studies mostly only included Autistic people (n=6), with only two studies including a non-Autistic comparison group.^41,43^ A total of 965 unique participants were included, with some overlap (n=6) in participants between two studies.^44,45^

We identified seven pieces of grey literature, six of which provided first-hand experiences of Autistic people in perimenopause. Six were from websites, with the seventh being a book chapter. Table 3 shows that there was limited detail regarding authors’ characteristics, diagnosis status, and any other factors that may have impacted on their menopause transitions. Both the peer reviewed (2020-2024) and grey literature (2013-2023) sources were published recently, highlighting the recency of content in this area.

### Convergent Integrated Synthesis

**Figure 2:**
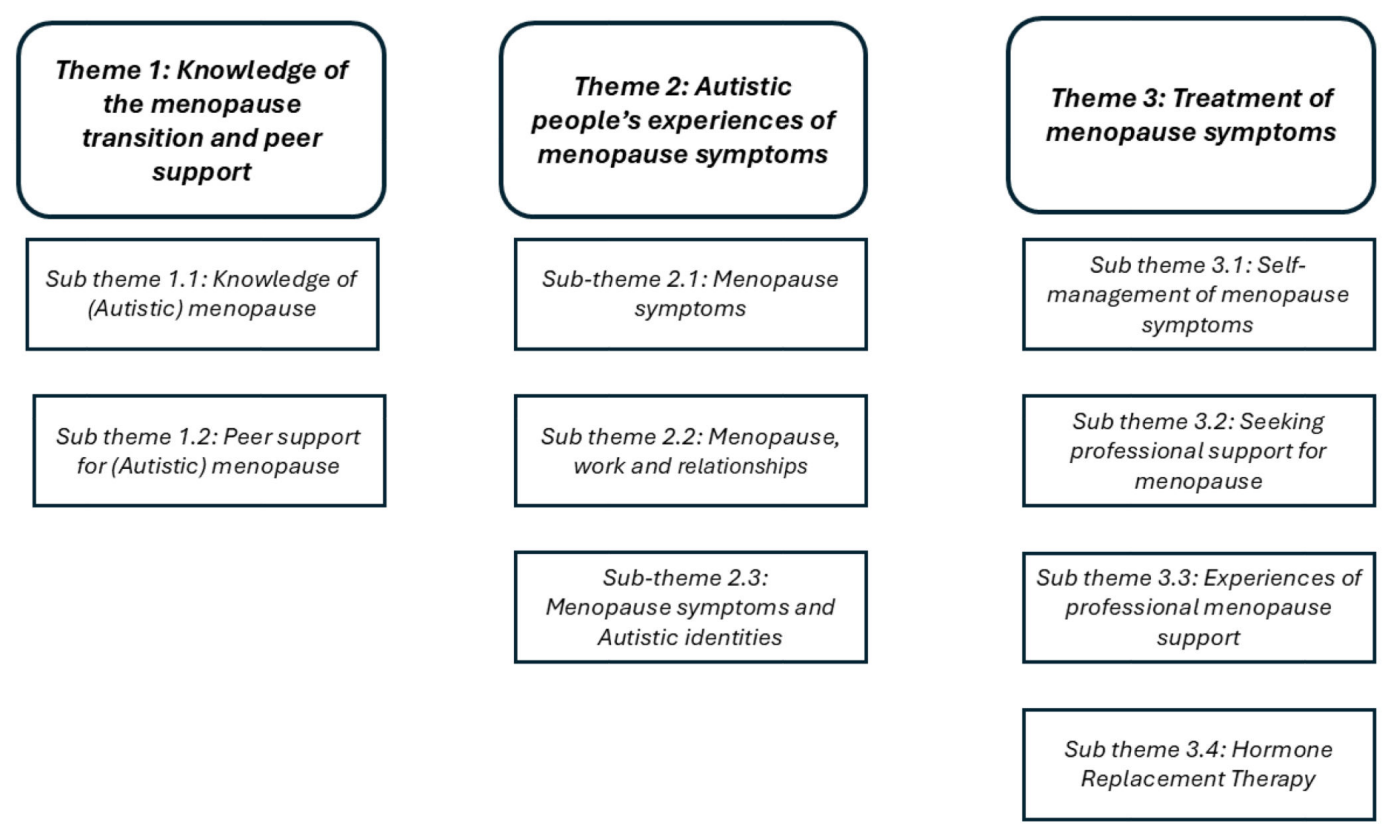
Graphical representation of convergent integrated synthesis

Our synthesis is comprised of three themes, with a range of sub-themes, which are represented in Figure 2. First, “knowledge of the menopause transition and peer support” focused on Autistic people’s knowledge of menopause symptoms generally and how they might impact Autistic people. It also included the importance of peer support for obtaining this knowledge. Second, our largest theme “Autistic people’s experiences of menopause symptoms” describes a broad range of symptoms, and their predictability, comparing these symptoms to non-Autistic people in two studies. It also outlines the impacts of symptoms on work, relationships and self-identity. Third, “Treatment of menopause symptoms” describes a range of medical and non-medical approaches to menopause management. We use the terms “Autistic people’s menopause transition” and “Autistic menopause symptoms” for brevity throughout *to refer to the experiences and symptoms experienced by Autistic people when they are going through the menopause transition, including perimenopause, menopause and post-menopause*.

### Theme 1: Knowledge of the menopause transition and peer support

This theme is focused on knowledge relating to the menopause. Overall, knowledge was extremely lacking and this could result in confusion and stress, for example, one participant having no idea their periods may stop due to menopause and worrying that they may have been pregnant.^46^ Online searching and associated peer support increased knowledge. The theme is divided into two sub-themes: *Knowledge of (Autistic) menopause*, and *Peer support for (Autistic) menopause*.

#### Sub theme 1.1: Knowledge of (Autistic) menopause

It was commonly reported that Autistic people had a lack of knowledge about the menopause transition in general.^38,40,44–47^ Sources noted Autistic people feeling that there was a lack of general menopause resources,^44^ that there was conflicting information available,^46^ and for those who were not cis-gendered, the focus on “women” in menopause resources could be jarring.^40^ In one survey, a large majority felt that they did have sufficient information overall, but there were many specific topics where they felt they lacked information, including the most noted topics: Autism and mental health impacts of the menopause.^40^ Other sources corroborated this finding that there was a lack of Autism-specific information and knowledge.^38,40,44,48–50^ This meant that some Autistic people did not know that menopause could be different for Autistic people,^40,48^ whilst others noted that regardless of knowledge, it would be hard to imagine how symptoms would impact them individually in advance.^40^ For some, this lack of information was attributed to both menopause and Autism being topics that were stigmatised and not openly discussed in society.^38,45,45^ However, it was noted by Autistic people with lived experience that it was important to have Autism-specific menopause resources.^38,44^

Lack of knowledge about the menopause led to most participants in a survey having unexpected symptoms.^40^ Furthermore, lack of knowledge about menopause was correlated with significant increases in menopause symptom difficulty,^40^ alongside feelings of confusion,^45^ uncertainty,^38,46^ worry and disempowerment.^38^ By contrast, understanding symptoms as part of the menopause was associated with less menopause symptom difficulty,^40^ and self-compassion.^51^

As a result of a lack of readily available information, including from health professionals,^40,44,46^ many participants spent time doing research on the menopause,^38,44,46,50,51^ which could be extensive.^38^ Sources of information about the menopause in general, or Autistic people’s menopause transition specifically, included the internet and social media,^40,46^ including support groups,^40^ and Autism conferences.^44^ People searched for information because they were beginning to experience^50^ or feeling worried about symptoms,^46^ and to use menopause symptom checkers.^51^

#### Sub theme 1.2: Peer support for (Autistic) menopause

Knowledge of Autistic people’s menopause transition was gained by talking online with other Autistic people, ^38,40,44–46^ including sharing practical strategies.^38,46^ It was noted that online discussions could be positive,^38,45,46^ including in terms of providing Autism-specific information,^45^ reducing isolation, and providing a sense of understanding and relief.^38^ However, limitations of peer support included lack of access to relevant online spaces for some,^44,46^ frustrating communications with others in these spaces,^46^ and relevant material not being accessed by those who did not yet know they were Autistic.^44^ One source noted the importance of providing group support on Autistic people’s menopause transition.^38^

Non-Autistic friends and family provided knowledge and support to some Autistic people,^40,45,46^ although differences in menopause symptoms could cause confusion.^45^ Some people had nobody to discuss the menopause with,^40,45^ including because it was difficult to discuss a taboo topic like menopause with those they were not close to.^44^

### Theme 2: Autistic people’s experiences of menopause symptoms

The largest theme, *Autistic people’s experiences of menopause symptoms*, focuses on a broad range of symptoms and the impacts of this in terms of occupational roles and identity. It is divided into three sub-themes: *Menopause symptoms; Menopause, work and relationships; Menopause symptoms and Autistic identities*.

#### Sub-theme 2.1: Menopause symptoms

In this sub-theme, we order symptoms in relation to how many sources they were mentioned within, although this is not a marker for how much data were included; for example, whilst vasomotor symptoms were reported in nine sources, there was little depth to the data. Overall, there was a lack of quantitative evidence to compare the severity of symptom experiences, however, there were many first-hand reports of symptoms being extremely challenging to manage.

##### Mental health, meltdowns and irritability

In a survey, around three quarters of participants reported changes in their mental health, around half reported changes in mood, over a third had reduced self-esteem, whilst a minority had increased self-esteem.^40^ In many sources, it was reported that some participants had pre-existing mental health difficulties prior to menopause,^40,44–46,48,52^ including anxiety and depression.^45,46^ For these, mental health challenges could increase during menopause,^44,45,48,52^ including because of experiencing menopause-induced palpitations,^45^ and nocturnal symptoms.^48^ Others reported new mental health changes, ^38,40,44–46,50,52,53^ including it being harder to regulate emotions, ^38,40,44,46,50,52,53^ mood swings,^47,50,53^ suicidality,^38,40,44^ tearfulness,^46,47^ panic attacks,^44^ overwhelm,^38,47^ and – following menopause – feeling dissociated.^46^ For some, the menopause transition lowered their threshold to have a meltdown,^44,46,51^ with reports of more frequent,^44,46^ or extreme, meltdowns, including violence and self-injury.^44,45^

##### Cognition and fatigue

During the menopause transition memory and concentration were impacted for around three quarters of participants in one survey.^40^ Brain fog was commonly reported,^38,40,45,46,53^ as was an increase in executive functioning challenges,^40,45,47,53^ and confusion.^46^ It was reported that disturbed sleep had impacts for cognition and coping.^44^ Fatigue was another commonly reported menopause symptom.^44–46,50,51^ It was noted that whilst people had previously been able to push through fatigue, that was no longer possible,^45,46,50^ due to “crushing tiredness”^45^ and burnout.^38^ In response, some adopted new strategies,^45^ as everyday tasks became more challenging.^44^ Strategies included taking more recovery time,^46^ and depending more on others.^45^

##### Sleep

Two sources noted that Autistic people who already experienced sleep challenges may have increased challenges during menopause,^48,50^ whilst others thought they may be impacted less as they were used to insomnia.^50^ Overall, menopause-associated sleep disturbances were commonly reported,^40,44–48,50–53^ and in one survey, most participants reported disturbed sleep.^40^ Sleep was impacted by menopause-induced night sweats,^45,47,50–53^ anxiety,^48^ and vivid nightmares.^46^ Sleep hygiene was reported to be an ineffective strategy in one source, ^48^ whilst another reported melatonin helped with disturbed sleep.^50^

##### Vasomotor

In one survey, there was no difference between Autistic and non-Autistic people in relation to vasomotor symptoms.^41^ However, for Autistic people, vasomotor symptoms were lower in pre-menopause compared to menopause and severity of symptoms did not reduce post-menopause.^41^ Moreover, it was noted in another survey that most participants experienced hot flushes and night sweats.^40^ Hot flushes were commonly reported,^44–47,50,51,53^ and could be associated with a tingling feeling.^50^ They were particularly challenging for those with temperature sensitivity,^44^ and having a hot flush in public was associated with embarrassment.^45^ Night sweats were also reported regularly.^45,47,50–53^ Two sources reported palpitations.^45,52^

##### Sensory

It was commonly reported that sensory sensitivity increased during menopause.^38,40,44–47,50,51^ This included to smells,^45,51^ temperature,^46^ light, touch, and sounds.^45^ Some people reported new sensory sensitivities, including to food.^44^ Furthermore, some sources reported that (unspecified) interoceptive difficulties occurred during the menopause transition.^40,45,46^ Autistic people noted these sensory differences impacted on mental health, including: mood,^44,50^ feeling overwhelmed,^44^ and more frequent^44^ and severe^44,45,51^ meltdowns. Severe sensory-induced meltdowns included uncharacteristic behaviours including stripping off their clothing down to their underwear at work,^44^ and shouting abuse at strangers.^51^ It was also noted that sensory differences could impact communication,^44^ intimate relationships, sexual enjoyment, and ability to do self-care or run errands outside of the home.^45^

##### Somatic

In two surveys, it was found that Autistic people had significantly more somatic symptoms than non-Autistic people.^41,43^ Moreover, in another survey, over half of participants reported joint pain and over a third headaches.^40^ However, in the qualitative data, there were limited reports of somatic symptoms. This included reporting of headaches,^51^ migraines being exacerbated by menopause,^46^ and tingling in the head with hot flushes.^50^ Unspecified pain was reported in three sources,^38,46,48^ with another reporting “body pain”.^51^

##### Menstrual changes

Unpredictable menstrual cycles were frequently reported.^38,40,44–46,52,53^ This included having heavier menstrual periods, ^38,46^ shorter menstrual cycles,^46^ and abnormal bleeding.^40^ The unpredictability was unsettling for those who preferred routine.^44,46,53^ By contrast, the certainty of medically induced menopaused was appreciated,^46^ and the cessation of periods associated with menopause was viewed positively.^40,45,46^ In one source, it was suggested that menstrual tracking could help Autistic people understand their menopause symptoms.^38^

##### Social interaction, communication and masking

Communication was impacted by the menopause.^38,40,44,45^ This included feeling less capable of social interaction,^38,44,45^ such as communicating their own needs to others,^44^ and some participants who could usually speak having times when they were unable to speak.^45^ Alongside this, some participants noted they were less able to process verbal and non-verbal communication,^45^ including struggling to understand others due to auditory processing challenges.^38^ Moreover, some Autistic people who had previously masked found that this became impossible^38,40,44^ or more energy consuming due to the menopause.^44–46^ Reducing masking was a relief to some,^40^ associated with self-understanding of their neurodivergence,^51^ and was accompanied by (unspecified) health benefits.^46^ However, it could also result in negative changes to their self-identity,^38,46^ increased difficulty fitting in,^38,40^ including at work,^38^ increased misunderstandings when communicating,^38^ and feeling more socially awkward.^38^

##### Physical changes

Weight gain was commonly described,^40,46,47,52,53^ and in one survey over half of participants reported it.^40^ Other changes included changes to scalp hair,^40,53^ including thinning for over a third, ^40^ growing more facial hair,^47,53^ and changes to body hair.^46^ Furthermore, over a third of participants in one survey reported dry skin and looser teeth.^40^ These physical changes could impact self-esteem^45^ and fatigue negatively,^52^ although some participants reported reduced concerns with their appearance accompanying menopause.^46^

##### Urogenital

One survey reported no significant differences in urogenital symptom severity between Autistic and non-Autistic people, although Autistic people reported more symptoms.^43^ Another survey found urogenital symptoms were common for Autistic people, with over half experiencing a decreased libido, over a third having vaginal drying and urinary incontinence, and a minority having pain during sex and experiencing Urinary Tract Infections.^40^ Reports of urogenital symptoms were, however, limited in qualitative studies, with only one study noting urogenital symptoms could impact on participants’ sex lives.^45^

##### Comparing Autistic and non-Autistic menopause symptoms

Only two surveys with small samples provided comparative data,^41,43^ these reported that Autistic people had significantly more severe menopause symptoms than non-Autistic people.^41,43^ That said, in one small survey, there was more variation (but not significantly so) in menopause complaint severity in the Autistic group than the non-Autistic group, suggesting a wider range of experiences.^43^ In this study, increased menopause severity was identified in Autistic people in relation to psychological and somatic aspects of the menopause.^43^ Furthermore, in Autistic people, but not in non-Autistic peers, higher menopause symptoms were correlated with higher levels of depression and Autistic traits.^43^

#### Sub theme 2.2: Menopause, work and relationships

##### Work

Prior to perimenopause, some participants had been unable to complete educational qualifications, or faced work stress or financial instabilitiy.^38^ Others noted that being Autistic had previously been advantageous for work, but that this had stopped during perimenopause.^44^ Overall, during the menopause, it was frequently reported that work felt harder,^38,40,44–46,52,53^ for example, feeling drained by attending a team meeting.^52^ This was for a variety of reasons, including brain fog and cognitive changes,^38^ executive function challenges,^44,45^ and not being able to mask at work any longer.^38,46^ It was noted in one source that the change in performance at work could occur very suddenly, and be a very large change.^38^ For many, this meant it became harder, or even impossible, to function at work. One person reported receiving disability accommodations, stating that they did not have to mask in the work place.^46^ A minority of participants changed jobs to be able to work from home,^40^ changed career,^38^ or took less demanding jobs^45^ to cope, although, in some cases this led to a reduction in income and debt.^45^

##### Relationships

Menopausal symptoms impacted on relationships.^38,40,44–46,51^ It was reported that this was because of increased difficulty communicating,^44^ increased fatigue, anxiety, and emotional lability,^45^ and feeling misunderstood leading to avoiding social interaction.^40^ Romantic relationships were also impacted by changes to libido (lower and increased),^45^ pain,^44^ picking the wrong partner during menopause,^45^ and relationship breakdown.^40^ Relationships with friends and family were also impacted for some,^44,45^ at a time when many participants had ageing parents,^38,44,45^ family bereavements,^40^ significant childcare responsibilities,^38,45,48^, including for neurodivergent children,^45^ and older children leaving home.^44,45^ It was noted in one study that these life circumstances could also influence the severity and impact of menopausal symptoms, because they were stressful.^45^ Some participants also suggested the difficult life events could also make it harder to recognise symptoms as part of the menopause transition,^45^ as emotional responses to difficult events were viewed as reasonable and proportionate.^46^

#### Sub-theme 2.3: Menopausal symptoms and Autistic identities

##### Fluctuating symptoms

Changing and uncontrollable menopause symptoms were regularly reported.^38,40,44–47,50–53^ This included fluctuating symptoms which could feel overwhelming,^40,44,46,52^ in the context of routines being reassuring.^47^ The suddenness and unpredictability of symptom changes could also make Autistic people feel out of control in a negative way.^38,44–47,50–53^ This included feeling unable to plan around menstrual cycles,^52^ and the feeling that a medically induced menopause may thus return control.^46^

##### Contextualising symptoms in Autistic identities

Some participants felt that interpreting menopause symptoms was more challenging for those who did not yet know they were Autistic, particularly when existing coping strategies failed,^38,44,46,51^ or atypical menopause symptoms were experienced.^44^ This finding was also present in quantitative data, with menopause difficulty increasing significantly for those with less Autism knowledge.^40^ A lack of self-understanding could compound the impact of menopause,^38^ add to distress,^46^ reduce opportunities for self-compassion,^45^ and to develop new coping strategies.^38,44^ After participants came to understand that they were Autistic, they better understood their menopause experiences,^40^ were more self-compassionate,^45,51^ and felt better about the menopause transition.^44,46^ For some, the menopause transition and Autism diagnosis occurred around the same time.^40,50^

### Theme 3: Treatment of menopause symptoms

This theme focuses on the ways in which Autistic people self-managed or had medical management of menopause symptoms. It was generally reported that self-management was the first response to symptoms. However, this may not have been truly a choice, as many participants reported barriers to accessing healthcare generally, and in relation to menopause care. Overall, participants made significant efforts to reduce the severity of their menopause symptoms, including changing their lives significantly, and paying for private healthcare. The sub-themes are: *Self-management of menopause symptoms; Seeking professional support for menopause; Experiences of professional menopause support*; and *Hormone Replacement Therapy*.

#### Sub theme 3.1: Self-management of menopause symptoms

Often the first response to menopause symptoms was an increase in non-medical forms of symptom management.^40,45,46,48,50,51,53^ This primarily centred around prioritising their needs,^40,45,46,50,51^ which for some came from understanding their own needs better,^40,45,46^ and thus having increased self-compassion,^50,51^ and a change in priorities.^46^ Self-management strategies included setting boundaries, including removing themselves from toxic relationships.^40,45^ Autistic people also spent more time doing activities they enjoyed,^45^ and being alone.^40^ Other strategies included carefully managing energy,^46^ stricter use of routines,^40^ trying not to worry about small things,^50^ taking breaks,^50^ and resting.^51^ However, this change was not always reported positively. First, it was noted that some people might not be able to engage in self-management activities because of fatigue,^45^ or other responsibilities.^38^ Also, second, it sometimes meant accepting help from others, which could be challenging to their self-identity.^45^

Some participants reported trying to engage in “healthy life choices”.^45^ This included restorative exercise,^46,48,50,51,53^ such as meditation,^48,50^ yoga,^50,53^ pilates,^53^ strength training,^46^ and walking in nature.^51^ Other lifestyle changes related to improved diet,^50,51^ comfort eating,^52^ sleep hygiene,^48^ and strategies to manage hot flushes, like using fans and cooling towels and wearing layers of clothes.^51^ A few personal accounts mentioned using supplements.^50,53^

#### Sub theme 3.2: Seeking professional support for menopause

In one survey, only a small minority stated that they did not want to discuss the menopause with their doctor.^39^ However, not all participants decided to seek professional support. Some who did not try to access care reported that previous bad experiences^38,40,46^ – including being scared or distrustful of doctors,^38,40,45^ and misdiagnosis^38^ – informed this decision. Among those who were considering trying to access menopause care, some had low expectations based on these previous bad experiences.^44^ In one study it was noted that there was fear of potential social work involvement for their children if they received menopause care.^38^

It was widely reported that it was difficult for Autistic people to access healthcare.^38,40,44,46^ Some people who wanted support could not access it,^40^ and this had intersectional impacts.^40^ Reported barriers included needing to make phone calls to book appointments,^38,40^ and services being overstretched, resulting in long waiting times.^38^ Moreover, it was reported in two qualitative studies that there was little professional support available for menopause.^38,44^ This was somewhat supported, in a large survey: less than half of participants had accessed professional menopause support. Of those who had accessed support, three fifths had used public healthcare, but two fifths used private healthcare.^40^ A further qualitative study noted that some participants used private healthcare despite the high financial costs.^38^ One blog focused on neurodiversity coaching as a way to build new strategies during Autistic people’s menopause transition.^52^

One study asked participants how they would prefer to receive menopause health care; the majority preferred face-to-face appointments, but a sizeable minority preferred email.^39^ Within appointments, however, there was no preference as to if the patient or health professionals should instigate a discussion about the menopause.^39^

#### Sub theme 3.3: Experiences of professional menopause support

Reports of professional menopause support were largely negative.^38–40,44–46^ First, considering barriers within healthcare systems, systemic issues included lack of continuity of care,^40,46^ appointments feeling rushed^38^ and not providing space to encourage patients to share their needs,^39^ and long delays to see menopause specialists.^38^ Alongside this, it was reported that doctors did not have a good understanding of the menopause.^38–40,44,46^ This resulted in some participants being ignored, misunderstood, not listened to, or not believed,^38–40,44^ sometimes despite significant menopause symptoms,^38,40^ or patient attempts to educate clinicians, such as by bringing along information.^38,40^ In this context, participants received inadequate^45^ or conflicting^46^ information. Furthermore, it was felt that doctors did not understand Autism,^39^ or how it presents in AFAB people,^38^ meaning it could feel unsafe to share a diagnosis, resulting in needs not being met.^39^ Moreover, it was noted that health professionals did not understand Autistic people’s menopause transition.^38,39,44,46^ In one study, almost all Autistic people perceived that their primary care staff never seemed to know how Autism impacts on menopause experiences.^39^ In two studies, participants reported their menopause symptoms had been misdiagnosed as depression^46^ and a personality disorder.^45^ Second, considering communication, it could be difficult to describe menopause symptoms,^38,40,44,45^ including because of interoceptive differences and alexithymia.^38,44^ It was also noted that it could be awkward to discuss the menopause, compared to other “physical” symptoms.^39^

Positive aspects of care included having helpful discussions with health professionals, ^44,45,51,52^ including where new information was learned.^52^ One primary care doctor gave a participant longer appointments and wrote take home notes,^46^ and two sources noted that specialist menopause services made them felt listened to,^46^ or helped them understand their symptoms.^51^

#### Sub theme 3.4: Hormone Replacement Therapy

HRT was the main medication group discussed to relieve menopause symptoms. It was used by some participants in a handful of papers,^40,41,45,46,51^ with one paper’s question on HRT use only being answered by around one third of participants,^41^ and another noting it was only a “few” participants who had used HRT.^45^ Other sources did not ask participants if they took HRT,^43^ did not mention it being used in their reporting of first-hand accounts,^44,49,53^ or explicitly stated that none of their participants had tried HRT.^38^ Initial use of HRT was impacted by worries about taking the medication,^41,45,46,49,53^ including in the context of being hypermobile, and at increased risk of pelvic organ prolapse,^46^ insufficient information.^46^ Others reported health professionals refusing to give a prescription for HRT.^38,41^ One participant who tried HRT found the side effects to be more difficult to tolerate than their symptoms, so stopped taking it.^46^ Another noted a lack of co-ordinated follow up after being prescribed HRT.^40^

## Discussion

Our review identified 15 sources, including eight peer-reviewed studies. Participants were almost exclusively Autistic people with lived experience of the menopause transition. Our analysis was divided into three areas: knowledge, symptoms, and management. Each of these has important links to other bodies of literature, which we explore below.

Menopause was sometimes reported to be stigmatised or taboo, as has been found in an evidence review focused on a general population,^54^ which may have resulted in the lack of knowledge about menopause in general reported in the included sources. However, Autistic people often have reduced peer and familial networks^55^ resulting in a ‘hidden curriculum’ of menopause knowledge, as has been found in relation to menstruation more generally.^56^ In some sources, participants reported undertaking extensive research, including engaging in online spaces for those going through the menopause or Autistic people, and this reportedly increased their knowledge, as has been found in relation to informal post-Autism-diagnosis support.^57^ However, requiring online knowledge acquisition disadvantages those who cannot access these materials, including due to digital poverty, and struggling to engage with materials that do not meet individual needs, for example for those with intellectual disabilities. It was also noted that infighting could occur in these spaces, and thus the spaces may not feel safe for all, and some who are in need of support may find themselves banned.^58^ Research on breastfeeding has also shown that for Autistic people there can be a difference between ‘book learning’ and being able to apply those insights to understand and treat one’s own health problems.^33^ Moreover, for those who can engage in these spaces, the quality of information was unknown, and many participants felt it would be beneficial to gain information from health professionals.

The majority of symptoms reported appeared to be well aligned to research on menopause in a general population,^5,7,8^ although there was a small amount of research which suggested Autistic people may experience symptoms to a greater degree.^41,43^ However, we feel that there are some important additions. Whilst some sensory factors, particularly relating to temperature, are commonly found in the menopause symptoms of a general population, Autistic people reported increased sensory sensitivity in a range of areas, sometimes leading to frequent and severe meltdowns.^44,46,51^ Increasing sensory sensitivity has been reported during menstruation^56^ and pregnancy^59^ for Autistic people, so this is not surprising in the context of a major hormonal shift. Moreover, some Autistic people reported taking more time to rest and avoiding social situations, whilst others reported that they experienced periods of burnout. Whilst the literature did not explicitly talk about shutdowns, we believe that reports of withdrawing from public spaces may have been a similar protective mechanism to allow a level of functioning to continue.^60^

It was also found that having pre-existing knowledge of being Autistic could be important in understanding menopause symptoms, being kinder to yourself and developing a new identity, as has been reported more generally in research on late diagnosed Autistic women.^61^ This finding is particularly important in the context of the under-diagnoses of women,^20^ which may particularly impact older women due to socialisation,^62^ as many undiagnosed Autistic women may not recognise their neurology, reducing opportunities to prevent burnout. Where Autistic people reported non-medical ways of trying to manage menopausal symptoms, many of these aligned to traditional health promotion guidance, such as “sleep hygiene”, healthy diets and exercise such as yoga and Pilates. These may be inappropriate for Autistic people, who are known to have challenges impacting all of these areas, including greater likelihood of sleep problems^63^ and requiring sensory stimulation to sleep;^64^ a need for safe food, especially in times of distress;^65^ and are more likely to be hypermobile.^66^ Moreover, some Autistic people noted that they had previously thrived in the workplace, but were struggling significantly since menopause. It is well established that menopause is taboo in the workplace,^67^ and Autistic people are under-employed,^68^ and thus workplace supports to account for Autistic menopause symptoms may be an important diversity initiative.

Barriers to healthcare access and negative reports of treatment received were very common throughout the sources reviewed. Barriers included systemic issues, including lack of time and expertise in menopause and Autism, which have been widely reported elsewhere in a variety of healthcare contexts.^32^ Interpersonal aspects of patient/health professional communication were also frequently reported negatively, with Autistic people feeling that they were not listened to. A systematic review has identified that health professionals struggle with communication with Autistic people.^69^ This can be contextualised in relation to the double^70^ and triple^71^ empathy problems. In the double empathy problem, it is argued that in cross-neurotype communication, it is only Autistic, and not non-Autistic, people who are expected to change.^70^ This has been extended in the triple empathy problem, where the additional lens of patient/health professional adds an extra layer of complexity.^71^

Furthermore, some people reported that they had taken information to their appointment in order to try to show evidence of their symptoms or to request treatment, and this was not always well received, which has also been found in a general population, despite online searching being common ahead of requesting a healthcare consultation.^72^ Overall, access to HRT appeared to be relatively rare. This may be contextualised in the context of current concerns around the over prescribing of HRT, in the context of cancer risks associated with older medications.^73^ Unsurprisingly, it appeared that some of those who received medical care received private treatment outside of public healthcare systems, which is associated with increased control and decreased waiting times.^74^ We hypothesise that this was to better have their needs met during treatment. Overall, there was very limited content in the data about the intersectional impacts of the menopause for Autistic people, with a few mentions of how being more underserved could impact on treatment.^38,46^ There are known intersectional impacts on healthcare in a general population, for example within maternity care, Black people experience significantly greater adverse outcomes at every stage^75^ and LGBTQ+ people report discrimination.^76^ We believe these intersectional impacts are likely to also impact on Autistic people during menopause.^77^

### Recommendations

Based on the synthesised data, we propose the following targeted recommendations. It should be noted that the data they are built on is limited, and thus the recommendations are tentative and should be updated in the future.

Autistic AFAB people may benefit from:

- Learning about the menopause prior to experiencing symptoms (e.g.: around age 30), so that they can better recognise their own menopause symptoms.
- Knowing that there is variation in menopause experiences for Autistic people, including how many symptoms people have and the severity of the symptoms
- Knowing that for Autistic people sensory sensitivities and meltdowns may increase during the menopause transition.
- Knowing that many Autistic people reduce their activities during the menopause, to cope with symptoms and the accompanying exhaustion.
- Knowing that masking gets harder or impossible for many Autistic people during menopause.
- Communicating with other Autistic people about their menopause symptoms, as it can help them feel connected and less alone.
- Being kind and self-compassionate to themselves when they experience menopause symptoms and/or can not do everything they used to.
- Learning more about being Autistic.

Partners and those supporting Autistic people during the menopause transition should:

- Know that the Autistic person’s dysregulation may increase, so their capacity to mask and undertake their previous activities may be reduced.
- Consider proactively offering support.

Health professionals supporting Autistic people during the menopause transition should:

- Provide accessible care for Autistic people as standard, ideally allowing longer appointment times so they feel listened to.
- Know that Autistic people may bring information to appointments as a communication aid.
- Consider that Autistic AFAB people aged >40 years with a range of new symptoms may be going through the menopause.
- Know that dysregulation, sensory sensitivities and meltdowns can increase during the menopause.
- Have up to date knowledge on menopause and HRT guidelines.
- Know that there is currently no evidence to suggest that Autistic people should not be treated with HRT.
- Offer Autistic patients with menopause symptoms HRT in line with prescribing guidelines.

Policy makers should:

- Reduce barriers to diagnosis of Autism in adulthood for AFAB people, including around the time of the menopause transition.
- Reduce barriers to accessible healthcare for Autistic adults.
- Improve disability supports in the workplace for Autistic people, and know that greater supports may be required around the time of the menopause.
- Reduce digital poverty for Autistic people, including those with intellectual disabilities.

Researchers should undertake additional research on Autistic people, the menopause and:

- Intersectionality, including co-occurring intellectual disabilities and ADHD.
- HRT use.
- Carers of Autistic people’s views and experiences.
- Interventions to reduce symptoms and improve quality of life, including peer support.

### Strengths and limitations

Our review was undertaken by an entirely Autistic team, including researchers, those with lived experience of menopause, and a primary care doctor. However, our group did not include representation of Autistic people with co-occurring intellectual disabilities. It is the first review of Autistic people’s menopause transition, and contained only eight peer reviewed sources, which we supplemented with grey literature. Our searches were limited to English language, which may have excluded relevant studies, including from cultures where a more positive view of the menopause transition are held. Participants in the studies were primarily recruited online, including social media and email lists, and thus may not be representative of all Autistic people. Moreover, ethnicity was not reported in most studies, and where it was reported a very high proportion of participants were of white ethnicity,^39^ which may have accounted for the limited information provided in relation to intersectionality. Furthermore, no sources included those who provide support to Autistic people, meaning their perspectives are not included. There was limited evidence on pain, incontinence, other urogenital symptoms and their impact on people’s sex lives, which can be common in menopause, or the use of HRT. None of the studies included developing or evaluating interventions to make menopause easier for Autistic people.

## Conclusion

Evidence shows that Autistic people are under supported in relation to the menopause. Accordingly, there is a clear need for more tailored information and supports for Autistic people before and during the menopause transition. This should be co-developed with Autistic people and tested to make sure it is of good quality. Health and social care professionals also need more information on Autistic menopause symptoms, so they are better placed to identify the signs of menopause and provide support. Peer support models may be worthy of development and evaluation to address the current gap in menopause support provision. Further research should investigate the intersectional experiences of Autistic people’s menopause transition and Autistic people’s use of HRT.

## Figures and Tables

**Table 1:** Inclusion and exclusion criteria

**Inclusion criteria**	***Population***	Autistic People, including those who self-identify, or who are undergoing diagnosis. Those who support Autistic people in this area of health, including, but not limited to, health professionals, family, and friends.
	***Context***	No restrictions.
	***Phenomenon***	Perimenopause, menopause and post-menopause menopause-related symptoms and experiences. Menopause which may be natural or induced/surgical. In practice, we would expect to see at least a paragraph related to the phenomenon for a study to be included in the review.
**Exclusion criteria**	Items that did not meet the population and/or phenomenon restrictions. Published prior to 2003. No full text. Full text not available in English.	

**Table 2:** Characteristics of studies

First Author and year published	Setting					Study details							Study quality
	Year of data collection	Funder	Country	Identity first/person first language	Any other factors which may affect results	Aim	Recruitment	Participants	Research design (JBI)	Data Collection	Data Analysis	Words included	JBI score
Brady et al. (2024)	Not stated, but ethical approval in 2022	Not stated	Canada (n=13) & UK (n=11)	Identity first	Incentives provided. Questions provided in advance. Autistic team member present during data collection. Included self-identifying as Autistic Majority of participant: white and female.	Explore how Autistic people in the UK and Canada experience menopause, access service, support and information.	Social media via community research associates	24 Autistic people (self ID or diagnosed) who were navigating menopause or postmenopausal. Aged 40-71	Qualitative	Demographic and Autistic menopause questionnaire (n=23) Semi-structured online focus groups (n=16 participants) and online interviews (n=8)	Qualitative Reflexive thematic analysis.	2,780	Yes: 9 Unclear: 1
Charlton et al. (2024)	2023	Unfunded	Not stated	Identity first	Included self-identified Autistic people in the Autistic group; validated using RADDS 14. Study not specifically about menopause, so may have attracted a wide group of participants. Sample size was relatively large. Online survey may be inaccessible to some potential participants.	Examine rates of menopause symptoms in Autistic and non-Autistic people at different stages of menopause.	Autistica’s research network, Cambridge Autism Research Database, author’s contacts.	242 Autistic & 100 non- Autistic aged 40+ years at different stages of reproductive life (premenopausal - postmenopausal)	Cross sectional	Online survey	ANOVA, chi-square &ANCOVA	1,074	Yes: 7 Unclear: 1
de Visser et al. (2024)	Not stated	Unfunded	United Kingdom	Identity first	Very broad range of ages; not all participants were menopausal. Sample was highly educated and white people were overrepresented. Many participants had other neuro- developmental disabilities	Explore Autistic people’s experiences of reproductive and sexual health in the context of primary healthcare.	Autistica’s email list (n=13,000), social media	136 Autistic adults aged 1871 years	Cross sectional	Online survey, using open and closed questions	Descriptive statistics, MANOVA and thematic analysis	1,889	Yes: 8
Groenman et al. (2022)	Not stated	Innovational Research Incentives Schemed VICI (NWO)	Netherlands	Mixture	Did not ask if HRT was used.	Explore: prevalence of PMDD; Autistic menopause symptoms during menopause transitions; relationship between menopause symptoms and Autism, anxiety, depression and ADHD	Mental health institutions; social media; Autism networks; researchers’ social networks.	Autistic people aged over 40; self-reported irregular or absent menstruation. **Autistic (n=30):** Diagnosed as Autistic; Excluded: intellectual disability, neurological disorders, alcohol or drug dependency. **Control (n=35):** Excluded: psychotic episodes, likely Autistic or ADHD	Cross sectional	Dutch version of the Menopause Rating Scale; Symptom Checklist-90 (SCL-90) subscales used for depression and anxiety proxy measure; ADHD self report (ASHD- SR); Autism Quotient (AQ)	*f-*test with group as independent variable and MRS score as the outcome. Bayseian analysis to assess group effects. Regression analysis to assess if MRS scores were associated with psychological symptoms.	318	Yes: 6 Unclear: 2
Jenkins et al. (2024)	2023	Social Sciences and Humanities Research Council of Canada	International, including: United Kingdom (n=211); United States (n=123); Canada (n=85); Europe (n=60)	Identity first	Few participants from the Global South; majority of participants were white; lack of accessibility to potential participants with learning disabilities; recall bias in some participants. Included those self-identifying. Autistic people involved in developing and undertaking the research.	To explore the support needs of Autistic people during menopause	Social media, researchers’ networks and support groups.	508 Autistic people with lived experience of menopause transition.	Cross Sectional	Online survey	Descriptive statistics, ANOVA. Reflexive Thematic Analysis	3,624	Yes: 8
Karavidas and de Visser (2021)	Not stated	Not stated	UK	Identity first	Offered in person or Skype interview mode. Included self-identifying as Autistic. Did not collect ethnicity or HRT use. Participants were “expert” in Autism; may be unrepresentative. One participant was 16 years post menopause ‘change’.	To understand Autistic people’s understandings of the menopause change.	Social media advertisement.	7 peri- (n=3) or post (n=4)- menopausal Autistic AFAB people who did not have intellectual disabilities. Aged 39-63. 6 participants = formal Autism diagnosis.	Qualitative	Semi-structured interviews via Skype. Participants were able to review and amend their transcript.	Material-discursive- intrapsychic approach within a critical realist epistemology, informed by thematic decomposition	3,858	Yes: 10
Moseley et al. (2020)	Not stated, but appears to be 2019 (Moseley et al., 2021)	Bournemouth University	International: British (n=4), South African (n=1) Australian (n=1) Unknown (n=1)	Identity first	Questions provided in advance. Small sample; did not ask if HRT was used; may be unrepresentative of Autistic community.	Explore: knowledge, difficulties and support needs relating to menopause.	Two Autistic- run Facebook groups for Autistic adults	7 Autistic (including self identifying) AFAB people going through the menopause aged 49-63 years.	Qualitative	Online focus group. AQ and RAADS- 14 used to assess Autism status.	Thematic analysis	1926	Yes: 6 Unclear: 3 No: 1
Moseley et al. (2021)	2019	Bournemouth University	International: British (n=13) Canadian (n=2) South African (n=1) Australian (n=1)	Identity first	Questions provided in advance; range of interview modes provided to increase comfort. Did not ask about mental health diagnoses; may be unrepresentative of Autistic community; recall bias (1 participant 21 years since last period). Includes six participants from Moseley et al. (2021).	Explore: menopause awareness, experiences and impacts, support needs.	Two Autistic- run Facebook groups for Autistic adults	17 Autistic (including selfidentifying) participants who believed they were going through the menopause (n=6) or had gone through it (n=11). 16 were cis women, aged 41-66 years.	Qualitative	Qualitative interviews, meeting participants access needs, including a/synchronicity AQ and RAADS- 14 used to assess Autism status.	Inductive thematic analysis guided by Interpretative Phenomenological Analysis principles.	2184	Yes: 7 Unclear: 2 No: 1

**Table 3:** Characteristics of gray literature

Author/Organisation and date published	Source Information				Author/subject information			
	Country	Content type	Type of data	Identity first or person first language	Characteristics of author/subject	Self-identified or diagnosed Autistic	Perimenopause, menopause or post-menopause?	Any other conditions?
Elcheson and Cook (2018)	Not stated	Book	First hand account with recommendations for Autistic people	Mixed	Not stated	Not stated	Perimenopause	Not stated
Kim (2013)	Not stated	Website	First hand account	Mixed	Self employed since 18; married for 25 years; non- Autistic family	Likely diagnosed, but unclear: “I was 42 when I discovered that I have Asperger’s Syndrome.”	Perimenopause	Not stated
LeafSoup (2023)	Not stated	Blog	First hand account	Mixed (including functional labels)	A mother and costume designer; enjoys drinking tea.	Not stated	Perimenopause	Not stated
Leveroy (no date)	UK	Website	Second-hand reporting of first hand experience	Mixed	Sales manager, age 47	Not stated	Perimenopause	Not stated
Noble (2023)	Not stated	Website	First hand account	Identity first	Not stated	Not stated	Perimenopause	Fibromyalgia, chronic fatigue, dysautonomia, ADHD.
Renton (2017)	Not stated	Website	First hand account with recommendations for Autistic people	Mixed (including functional labels)	Journalist and filmmaker	Unclear “with Aspergers”	Perimenopause	Not stated
Silber (2017)	Not stated	Website	First hand account	Identity first	Not stated	Not stated	Perimenopause	Not stated

## Community Brief

### Why is this an important issue?

Menopause is when menstrual periods stop forever. The menopause transition usually happens around the age of 45-55 years. People usually have symptoms for several years *before* the menopause; symptoms can have a big impact on people’s lives.

### What is the purpose of the review?

To bring together all the existing research to better understand the menopause-related experiences and needs of Autistic people.

### What did the authors do to review the literature?

We did a systematic literature search and found first-hand accounts, like blogs, which focused on Autistic experiences of menopause.

### What studies did the authors find?

Our search found eight studies and seven other first-hand accounts. Most of the sources only included Autistic people. Two studies had some non-Autistic participants as a comparison group.

### In summary, what did those studies show?

We developed three themes.

First, “knowledge of the menopause transition and peer support”. Most Autistic people didn’t know much about menopause symptoms when they started having them. This included the range of menopause symptoms that Autistic people could have. Also, some Autistic people used online forums and groups to find out more about what the menopause is like for Autistic people.

Second, “Autistic people’s experiences of menopause symptoms” describes lots of symptoms:

- mental health, meltdowns and irritability (found in 11 sources);
- cognition, fatigue and functioning (found in 10 sources);
- sleep (found in 10 sources);
- hot flushes and night sweats (also called “vasomotor” symptoms; found in 9 sources);
- increased sensory sensitivity (found in 8 sources);
- pain and headaches (also called “somatic” symptoms; found in 8 sources);
- changes to periods (found in 7 sources);
- changes to how they communicated, including masking (found in 6 sources);
- changes to their weight, hair, skin and teeth (found in 6 sources); and
- changes to their genitals and peeing (called “urogenital” symptoms; 3 sources).

Two studies found Autistic people had worse menopause symptoms than non-Autistic people. Autistic people said menopause symptoms impacted on work and relationships. They also changed how people felt about themselves.

Third, “treatment of menopause symptoms” describes non-medical ways people tried to feel better, like taking more rest. It also contains experiences of seeking medical help. Most people who saw a health professional said it was a bad experience for them. Not many people had tried Hormone Replacement Therapy (also called “HRT”).

### What are the remaining gaps in the literature?

There wasn’t much information about:

- how menopause impacted underserved parts of the Autistic community (“intersectionality”)
- urinary incontinence (peeing yourself) and other urogenital symptoms, which can be common in menopause.
- Using HRT

There weren’t any studies which tested tools or approaches to make menopause easier for Autistic people.

### Based on this review, what do the authors recommend?

Autistic people need better information and tools to prepare for, and during, the menopause transition. This should be developed with Autistic people and tested to ensure it is good quality; peer support models may be worthy of development and evaluation. Health professionals also need more information on Autistic people’s menopause transition.

## Footnotes

* Please note, we have chosen to capitalise Autism and Autistic in this review as a marker of identity, culture and community.^19^
